# A Comparative Study of Prevalence of RTI/STI Symptoms and Treatment Seeking Behaviour among the Married Women in Urban and Rural Areas of Delhi

**DOI:** 10.1155/2015/563031

**Published:** 2015-01-27

**Authors:** Anjana Verma, Jitendra Kumar Meena, Bratati Banerjee

**Affiliations:** Department of Community Medicine, Maulana Azad Medical College, New Delhi 110002, India

## Abstract

*Background*. In developing countries, women are at high risk for several reproductive health problems especially RTI/STIs. Since all RTIs/ STIs are preventable and most of them are curable, it is pertinent to study the determinants of the health seeking behaviour. *Objectives*. To compare the prevalence and treatment seeking behaviour about RTI/STI symptoms among the married women of reproductive age group (18–45 years) living in urban and rural area of Delhi. *Methods*. A cross-sectional study was done among the married women of reproductive age group residing in Pooth Khurd, a village in North West district of Delhi, and Delhi Gate, an urban locality situated in central Delhi. *Results*. In this study, the prevalence of RTI/STI symptoms was found to be similar in both urban (42.3%) and rural area (42%). In urban area, 73% sought treatment, while in rural area only 45.6% sought treatment. Prevalence of the symptoms was found to be higher among the study subjects who were not using any contraceptive method, had history of abortion, and were with lower educational status, in both urban and rural areas. Treatment seeking behaviour was significantly higher among the educated women, contraceptive users, and older age group women in both rural and urban area.

## 1. Introduction

Some 340 million new cases of curable sexually transmitted infections (STIs) occur every year. The figure does not include HIV or other viral STIs like hepatitis B, genital herpes, and genital warts, which are not curable. The most common of the curable STIs are gonorrhea, syphilis, chlamydia, and trichomoniasis. Sexually transmitted infections constitute a significant health burden and increase the risks of transmission of HIV [1]. Reproductive tract infection (RTI) is a global health problem among women, living in South East Asian Region (SEAR) countries. Studies have found the prevalence of RTI in India, Bangladesh, Egypt, and Kenya is in the range of 52–90 per cent. More than a million women and infants die of the complications of RTI every year [2]. Reproductive tract infections (RTIs) are caused by organisms normally present in the reproductive tract or introduced from the outside during sexual contact or medical procedures. These different but overlapping categories of RTI are called endogenous, sexually transmitted infections (STIs), and iatrogenic, reflecting how they are acquired and spread [3].

The prevalence of self-reported RTI symptoms among Indian women has been found to be 11–18% in nationally representative studies [4, 5] and 40–57% in various other studies [6–8], while the prevalence of laboratory-diagnosed RTIs has ranged from 28% to 38% [9, 10]. According to studies that have explored women's patterns of seeking treatment for RTI symptoms, between one-third and two-thirds of symptomatic women did not seek treatment [6, 8–10].

## 2. Materials and Methods

### 2.1. Study Design

It is a community based cross-sectional study.

### 2.2. Study Area

The study area is Pooth Khurd, a village in North West district of Delhi, and Darya Ganj, urban locality in central Delhi.

### 2.3. Study Population

Married women of reproductive age group residing in Pooth Khurd and Darya Ganj area of Delhi are the study population.

### 2.4. Study Period

The study was carried out from January 2012 to May 2013.

### 2.5. Sample Size Calculation

Taking the prevalence of RTI symptoms among married women (15–44 years) as 37% (RCH-II survey), with relative error of 20%, and taking nonresponse rate of 10%, total sample size is calculated to be 215, each in rural and urban area.

### 2.6. Methodology

Sampling was done using the simple random sampling technique. A sample of 215 women was selected using the random number table from the list of eligible couples noted in the registers at health centres serving these areas. In case the subject was not found eligible for the study (residing in the area for less than 6 months), then the woman listed next in the eligible couple register was selected. Investigation was carried out by the investigator, who was pursuing the postgraduation in community medicine in the duration of study period, by paying house to house visits. During the house visits, investigator introduced herself to the eligible women, explained the purpose and procedure of the study, and ensured complete confidentiality of their responses. In this study, the mean age of the study subjects was 31.3 years in urban and 28.4 years in rural area. Most of the study subjects were between the age of 26 and 35 years in both urban and rural area.

### 2.7. Study Tools

A predesigned, pretested, and semistructured questionnaire was used to take the interview of eligible women. The questionnaire had both open and closed ended questions. Questionnaire included following parts:

- questions to assess sociodemographic profile of the subject, identification data, namely, age, address, religion, occupation, socioeconomic status, and so forth;
- questions about the menstrual history of the women;
- questions about the obstetric history;
- questions to assess the knowledge regarding RTIs and history of occurrence of any RTI symptom in the past 3 months;
- questions to assess the treatment seeking behaviour for RTI symptoms.

### 2.8. Ethical Issues

The aims, objectives, and procedure of the study were explained to all the women. Informed consent was taken from all the participants. Complete confidentiality regarding patient information was maintained through all the stages of the study.

### 2.9. Statistical Tests

Data was analysed using SPSS version 17. Percentage and proportions were calculated for prevalence of the symptoms and treatment seeking behaviour. Chi square and Fisher Exact test were used as tests of significance in univariate analysis. A *P* value of less than 0.05 was considered significant.

## 3. Results

In this study, the prevalence of RTI/STI symptoms was found to be similar in both urban (42.3%) and rural area (42%). In urban area, 73% sought treatment, while in rural area only 45.6% sought treatment.  Table 1 shows the sociodemographic characteristics of the respondents.

Table 2 shows various obstetric and behavioural characteristics of the respondents. 39% of study subjects in urban area and 37.2% in rural area had history of abortion. Out of these, induced abortions comprised 81% in urban and 79% in rural area. Most of the women in both urban and rural area were using sanitary pad as method of menstrual hygiene. 76% of study subjects in urban and 51.6% in rural area were using some method of contraception. Condom was the most common method of contraception followed by tubectomy in both urban and rural area.

When asked about the knowledge of RTI/STI symptoms, 50.2% of respondents in urban and 41.8% in rural area were aware about the presence of discharge as an indication for infection. Only 20% of respondents in urban and 10% in rural area had heard of lower abdominal pain as a symptom of RTI/STI. Very few respondents knew about other symptoms of RTI/STI such as dyspareunia, dysuria, burning micturition, infertility, and genital ulcer. When participants were asked about the possible ways of contracting the disease, 34.9% of women living in urban area and 20% of rural women replied that disease can be acquired from infected partner. Poor genital hygiene was reported to be the cause of RTI/STI symptoms by 25% of urban respondents and 37% of rural women. In this study the prevalence of RTI/STI symptoms was found to be similar in both urban (42.3%) and rural area (42%). Vaginal discharge (77.8%) was reported as the most common symptom by the rural women followed by lower backache (51%) and lower abdominal pain (25.6%), whereas in urban area lower backache (63%) was the most common symptom followed by lower abdominal pain (49%) and vaginal discharge (35%) (Table 3).

Table 4 shows that, out of the 91 symptomatic women in urban area, 66 (73%) sought treatment, 46 (70%) consulted private practitioner, and 20 (30%) went to a government hospital. 92% of the treatment seeking women were compliant to the treatment. In rural area, out of the 90 symptomatic women, 41 (45.6%) sought treatment, 21 (51%) consulted private practitioner, and 20 (49%) went to a government hospital. 90% of the treatment seeking women were compliant to the treatment in rural area.

Table 5 shows the prevalence of RTI/STI symptoms in relation to some sociodemographic, obstetric, and behavioural factors in urban and rural Delhi. Based on the presence of one or more symptoms, the prevalence of RTI/STI symptoms was found to be 42.3% in urban area. It was highest in the 18 to 25 years of age group than other age groups; however the difference was not statistically significant. The prevalence of RTI/STI symptoms was found to be highest among the illiterate respondents (54%) and decreases significantly with increasing level of education (*P* value = 0.03). There was no significant difference in the prevalence of symptoms due to occupation, per capita income, menstrual hygiene practices, and parity. Contraceptive users had lesser prevalence (38.6%) of RTI/STI symptoms as compared to nonusers (54%) and the difference was statistically significant (*P* value = 0.021). The prevalence was highest (56%) in those who had history of abortion and (35%) in those with none of the risk factors of abortion or inserted IUCD (*P* value = 0.04).

In rural Delhi, the prevalence of RTI/STI symptoms was 42.3%. It was highest in the 18 to 25 years of age group than other age groups; however the difference was not statistically significant. The prevalence of RTI/STI symptoms was found to be highest among the respondents who were educated up to high school (45%) and was lowest among the respondents who were post-high school educated (*P* value = 0.046). There was no significant difference in the prevalence of symptoms due to occupation, per capita income, and menstrual hygiene practices. The prevalence of symptoms was found to be significantly associated with parity (*P* value = 0.009). Contraceptive users were found to have lesser prevalence (35.1%) of RTI/STI symptoms than nonusers (49%) and the difference was statistically significant (*P* value = 0.029). The prevalence was highest (54%) in those who had history of abortion and (30%) in those with CuT insertion (*P* value = 0.04).

Table 6 shows the treatment seeking behaviour of the symptomatic women in relation to some sociodemographic, obstetric, and behavioural factors in urban and rural Delhi. In urban area, it was found that treatment seeking behaviour increased with age (*P* value = 0.001) and education status (*P* value = 0.003) of the study subjects. Contraceptive users had higher percentage of treatment seeking behaviour as compared to the nonusers with a significant *P* value of 0.001. Treatment seeking behaviour was higher among the women with higher per capita income, but this difference was not statistically significant. Similarly, in rural Delhi (Table 6), treatment seeking behaviour was found to be associated significantly with higher age (*P* value = 0.001), higher education (*P* value = 0.003), and contraceptive usage (*P* value = 0.001). However, treatment seeking behaviour was also found to be associated with the occupational status of the women. Working women had higher percentage of seeking treatment as compared to housewives with statistically significant difference (*P* value = 0.04).

## 4. Discussion

### 4.1. Prevalence of RTI/STI Symptoms

In this study the prevalence of RTI/STI symptoms was found to be similar in both rural (42%) and urban (42.3%) area of Delhi. This is higher than that reported in RCH-II survey (2002–04), according to which the prevalence of RTI/STI symptoms was 39.3% in rural and 33.6% in urban women [11]. In a study conducted by Kosambiya et al. in Surat, the prevalence of RTI/STIs was higher in urban (69%) than rural area (53%). The higher prevalence among urban population was attributed to greater proportion of migratory population residing in urban areas of Surat [12].

Vaginal discharge (77%) was reported as the most common symptom by the rural women followed by lower backache (51%) and lower abdominal pain (25.6%), whereas in urban area lower backache (63%) was the most common symptom followed by lower abdominal pain (495) and vaginal discharge (36%).

### 4.2. Knowledge about RTI

When asked about the knowledge of RTI/STI symptoms, 50.2% of respondents in urban and 41.8% in rural area were aware about the presence of discharge as an indication for infection. 20% of respondents in urban and 10% in rural area had heard of lower abdominal pain as a symptom of RTI/STI. This is much higher than reported in another study done by Kosambiya et al. in Surat, in which only 14% of the respondents were aware about the presence of discharge as an indication of infection and 9% knew lower abdominal pain as the symptom of RTI [12]. Very few respondents knew about other symptoms of RTI/STI such as dyspareunia, dysuria, burning micturition, infertility, and genital ulcer. When participants were asked about the possible ways of contracting the disease, 34.9% of women living in urban area and 20% of rural women replied that disease is acquired from infected partner. This finding is similar to that reported in Surat, in which 22% of rural and 41% of urban area replied infected partner is the source of infection [12]. Unhygienic conditions were reported to be the cause of RTI/STI symptoms by 37% of rural women and 25% of urban respondents.

### 4.3. Factors Associated with RTI Symptoms

The prevalence of RTI/STI symptoms was found to be associated with education in both urban and rural areas, which decreased significantly with increasing level of education (*P* value < 0.05). This finding is comparable to a study carried out in Rajasthan by Bansal et al. in which RTI was highly prevalent among illiterates (62.5%) as compared to literates (48.3%). Number of RTI cases decreased with an increase in educational status, showing a direct relationship between the two [13]. The prevalence of symptoms was found to be significantly associated with parity (*P* value = 0.009) in rural area. The prevalence of symptoms was found to be lowest among the nulliparous and highest among the women with parity of four or more. This finding is comparable to a study done by Rani et al. in Gorakhpur, which revealed that overall prevalence of RTIs was maximum (42.0%) in women who were having five or more children and minimum (34.2%) in women who had one or no child. This difference was statistically significant (*P* value = 0.01) [14]. Similar finding was reported in a study done in Ludhiana by Philip et al. in which it was found that the prevalence of the symptoms increased with parity, with the prevalence being lowest (7.7%) in the nulliparous and highest (25.0%) in the multiparous with parity >4. The prevalence was 16.8% in those with parity 1-2 and 18.3% in those with parity 3-4 [15].

Contraceptive users were found to have lesser prevalence of RTI/STI symptoms than nonusers and the difference was statistically significant (*P* value = 0.021) in both urban and rural areas. The prevalence was highest in those who had history of abortion in both urban and rural areas. Similar findings were reported by Rani et al. [14] and Philip et al. [15], in which prevalence was highest among the study subjects who had history of abortion. This can be attributed to the fact that operative procedures make women more prone to ascending infections.

There was no significant difference in the prevalence of symptoms due to occupation, per capita income, and menstrual hygiene practices.

### 4.4. Treatment Seeking Behaviour

In the present study, out of the 91 symptomatic women in urban area, 66 (73%) sought treatment, 46 (70%) consulted private practitioner, and 20 (30%) went to a government hospital, while 25 (27%) did not seek any treatment. This proportion is much higher than reported by a study done in Ludhiana by Philip et al., in which the majority (64.4%) of the respondents with symptoms did not seek any treatment. The preferred source of treatment was a private practitioner, followed by “desi” medicines [15]. This can be attributed to the differences in education status and health awareness among the study subjects living in Delhi and Ludhiana.

In rural area, out of the 90 symptomatic women, 41 (45.6%) sought treatment, 21 (50%) consulted private practitioner, and 20 (49%) went to a government hospital, while 49 (54.4%) did not seek any treatment. This proportion is higher than reported in a study done by Aggarwal et al. in a rural area of Dehradun, in which 36.4 per cent of the respondents responded that they would like to visit an STD specialist and 19.3 per cent expressed that they would consult an MBBS doctor. 5.7 per cent of the symptomatic women still wanted to be treated by quacks and 4.5 per cent wanted to take treatment from other traditional healers [16]. In a study done by Ravi and Kulasekaran in rural area of Tamil Nadu it was revealed that 80.7% of the women sought treatment [17]. This is much higher than found in present study. This difference can be because of the differences in sociodemographic characteristics of the populations.

In urban area, out of 91 symptomatic women, 66 (73%) sought treatment, 46 (70%) consulted private practitioner, and 20 (30%) went to a government hospital. 92% of the treatment seeking women were compliant to the treatment. Easy accessibility to the private practitioners can be the possible reason for the preference of private practitioner for seeking treatment. In rural area, out of the 90 symptomatic women, 41 (45.6%) sought treatment, 21 (50%) consulted private practitioner, and 20 (49%) went to a government hospital. This finding is similar to other studies, in which the majority of women had consulted private providers, suggesting that a preference for these providers persists despite the numerous initiatives undertaken by the Indian government to improve access to sexual and reproductive health services in the public sector [8, 9, 18–20].

### 4.5. Factors Associated with Treatment Seeking Behaviour

In present study, treatment seeking behaviour increased with educational status of the study subjects in both urban and rural area. This finding is similar to that reported in a study done by Ravi and Kulasekaran in Tamil Nadu, in which overwhelming proportion of women received treatment of STIs who completed secondary education (85.7%) compared to those who completed primary education (70.0%) and illiterates (66.7%) [17]. Similarly in other studies, schooling was associated with treatment seeking behaviour of the study subjects [10, 18], from formal providers, likely because young women with more schooling had more information about protective actions and the resources to adopt them than did others [18].

In our study treatment seeking behaviour was found to be associated with older age as compared to younger women. This finding differs from study done by Ravi and Kulasekaran in Tamil Nadu, in which it was observed that younger women were much more likely to receive treatment for their STIs than the older women [17]. This can be explained because of the differences in sociodemographic profile and level of health awareness among the women of these two states. In our study older age group women were found to have better treatment seeking behaviour than younger women. This can be attributed to the fact that younger women do not have decision making power regarding seeking health services as compared to older age group women, because of various traditional cultural beliefs and attitudes of the people living in this area.

In present study, working women had better treatment seeking behaviour than nonworking women in rural area. This finding is comparable to that found in a study done by Sabarwal and Santhya in which the greater proportion of the working women sought treatment as compared to the nonworking women [18].

In present study, the contraceptive users had higher percentage of treatment seeking behaviour as compared to the nonusers, likely because contraceptive users had more information and awareness about health care services, due to regular visits to the health centre and communication with health workers.

## 5. Conclusion

In this study, it was found that the prevalence of RTI/STI symptoms is high in both urban (42.3%) and rural (42%) areas. Prevalence of the symptoms was found to be higher among the study subjects who were not using any contraceptive method, had history of abortion, and were with lower educational status, in both urban and rural areas. In rural area, in addition to above factors, prevalence of symptoms was found to be significantly associated with parity. The treatment seeking behaviour is better in urban area (73%), as compared to rural area (45.6%). However, in both areas, women preferred private practitioner for seeking treatment compared to government facilities, because of the long waiting time and unavailability of medicines in government health facilities. Treatment seeking behaviour was significantly higher among the educated women, contraceptive users, and older age group women in both rural and urban area.

There is a need to educate women about the symptoms of RTI/STI, their prevention, and the importance of timely treatment in both urban and rural areas. The availability of the RTI/STI treatment kits should be ensured in all the primary health centres to increase the usage of the government services.

## Figures and Tables

**Table 1:** Sociodemographic characteristics of the study subjects of urban and rural area (*n* = 215).

Characteristics	Urban *n* (%)	Rural *n* (%)
Age		
18–25	54 (25)	60 (28)
26–35	110 (51)	103 (48)
36–45	51 (24)	52 (24)
Educational status		
Illiterate	52 (24.2)	63 (29.2)
Primary	13 (6)	15 (7)
Middle	45 (21)	43 (20)
High school	43 (20)	41 (19.1)
Secondary	35 (16.3)	34 (15.8)
Graduate	20 (9.3)	15 (7)
Postgraduate	7 (3.2)	4 (1.9)
Occupation		
Housewife	200 (93)	198 (92.1)
Unskilled worker	4 (1.8)	6 (2.8)
Semiskilled worker	3 (1.4)	4 (1.9)
Skilled worker	2 (1)	3 (1.4)
Shopkeeper/clerical	3 (1.4)	2 (0.9)
Semiprofessional	3 (1.4)	2 (0.9)
Monthly per capita income		
Up to 1000	70 (32.6)	77 (35.8)
1001–2000	50 (23.3)	52 (24.2)
2001 or more	95 (44.1)	86 (40)

**Table 2:** Obstetric and behavioural characteristics of the study subjects (*N* = 215).

Characteristics	Urban *n* (%)	Rural *n* (%)
Parity		
0	2 (1)	23 (10.7)
1-2	101 (47)	138 (64.2)
3-4	107 (50)	50 (23.2)
>4	5 (2)	4 (1.9)
History of abortions		
Yes	84 (39)	80 (37.2)
No	131 (61)	135 (62.8)
Last abortion, how many years back		
≤1 year	12 (14.3)	8 (10)
>1 year	72 (85.7)	72 (90)
Type of abortion		
Spontaneous	16 (19)	17 (21)
Induced	68 (81)	63 (79)
Menstrual hygiene practices		
Ordinary cloth	54 (25)	69 (32.1)
Sanitary pad	129 (60)	111 (51.6)
Both sanitary pad and cloth	32 (15)	35 (16.3)
Usage of contraceptive methods		
Yes	163 (76)	111 (51.6)
No	52 (24)	104 (48.4)
Methods of contraception		
None	52 (24)	61 (28)
Pills	19 (9)	10 (4.7)
Condom	95 (44.2)	90 (42)
IUCD	6 (2.8)	20 (9.3)
Safe period	1 (0.5)	0
Withdrawal	10 (4.7)	0
Tubectomy	32 (14.8)	34 (16)

**Table 3:** Knowledge and prevalence of RTI/STI among study subjects.

Characteristic	Urban *n* (%)	Rural *n* (%)
Knowledge of symptoms^*^		
Discharge	108 (50.2)	90 (41.8)
Dyspareunia	4 (2)	2 (1)
Dysuria	9 (4)	4 (2)
Lower abdominal pain	43 (20)	22 (10)
Infertility	2 (1)	2 (1)
Burning micturition	9 (4)	6 (3)
Genital ulcer	2 (1)	2 (1)
How does one get the disease?		
Poor genital hygiene	54 (25.1)	80 (37)
Infected sexual partner	75 (34.9)	43 (20)
Do not know	86 (40)	92 (42.8)
History of symptoms experienced		
Yes	91 (42.3)	90 (42)
No	124 (57.7)	125 (58)
History of symptoms experienced^*^		
Discharge	32 (35)	70 (77.8)
Lower backache	57 (63)	46 (51.1)
Lower abdominal pain	45 (49)	23 (25.6)
Itching over vulva	15 (7)	5 (5.6)
Dyspareunia	0 (0)	2 (2.2)
Infertility	2 (2)	4 (4.4)
Genital ulcer	2 (2)	2 (2.2)
Burning micturition	18 (20)	10 (11.1)

^*^Mutually not exclusive.

**Table 4:** Treatment seeking behaviour of the respondents.

Characteristic	Urban *n* (%)	Rural *n* (%)
Treatment seeking symptomatic women	66 (73)	41 (45.6)
Government hospital	20 (30)	20 (49)
Private practitioner	46 (70)	21 (51)
Compliance to the treatment among women	61 (92)	37 (90)

**Table 5:** Prevalence of symptoms suggestive of RTI/STI in relation to sociodemographic, obstetric, and behavioural factors in urban and rural area.

Characteristics	Urban			Rural		
	Symptoms present *n* (%)	Total *n* (%)	*P* value	Symptoms present *n* (%)	Total *n* (%)	*P* value
Age						
18–25	29 (54)	54 (25)	0.710	32 (53.3)	60 (28)	0.622
26–35	53 (48)	110 (51)		48 (46.6)	103 (48)	
36–45	9 (17.6)	51 (24)		10 (19.2)	52 (24)	
Education						
Illiterate	28 (54)	52 (24)	**0.032**	27 (43)	63 (29.3)	**0.046**
Up to high school	40 (40)	101 (47)		45 (45.5)	99 (46)	
Post-high school	23 (37.1)	62 (29)		18 (34)	53 (24.7)	
Occupation						
Working	6 (40)	15 (7.5)	0.970	7 (41.2)	17 (7.9)	0.952
Housewife	85 (42.5)	200 (93)		83 (42)	198 (92.1)	
Monthly income per capita						
Up to 1000	30 (42.8)	70 (32.6)	0.124	28 (36)	77 (35.8)	0.121
1001–2000	21 (42)	50 (23.3)		28 (54)	52 (24.2)	
2001 or more	40 (42)	95 (44.1)		34 (40)	86 (40)	
Menstrual hygiene practices						
Ordinary cloth	23 (42)	54 (25)	0.198	32 (46.4)	69 (32.1)	0.201
Sanitary pad	58 (45)	129 (60)		48 (43.2)	111 (51.6)	
Both sanitary pad and cloth	10 (31.25)	32 (15)		10 (28.6)	35 (16.3)	
Parity						
0	0 (0)	2 (1)	0.124	4 (17.4)	23 (10.7)	**0.009**
1-2	43 (42.6)	101 (47)		60 (43.4)	138 (64.2)	
3-4	45 (42)	107 (50)		24 (48)	50 (23.3)	
>4	3 (60)	5 (2)		2 (50)	4 (1.8)	
Contraceptive usage						
Yes	63 (38.6)	163 (76)	**0.021**	39 (35.1)	111 (51.6)	**0.029**
No	28 (54)	52 (24)		51 (49)	104 (48.4)	
Gynaecological risk factors						
Abortion	47 (56)	84 (39)	**0.04**	43 (54)	80 (37.2)	**0.004**
CuT	0 (0)	6 (2.8)		6 (30)	20 (9.3)	
Nil	44 (35)	125 (58.2)		41 (35.7)	115 (53.5)	

**Table 6:** Treatment seeking behaviour in relation to some sociodemographic, obstetric, and behavioural factors in urban and rural area.

Characteristics	Urban (*n* = 91)			Rural (*n* = 90)		
	Sought treatment *n* (%)	Total symptomatic respondents *n* (%)	*P* value	Sought treatment *n* (%)	Total symptomatic respondents *n* (%)	*P* value
Age						
18–25	19 (65)	29 (32)	**0.001**	6 (18.8)	32 (36)	0.000
26–35	40 (75)	53 (58)		28 (58.3)	48 (53)	
36–45	7 (78)	9 (10)		7 (70)	10 (11)	
Education						
Illiterate	16 (59.3)	27 (30)	**0.003**	10 (37)	27 (30)	**0.04**
Up to high school	35 (78)	45 (50)		22 (48.9)	45 (50)	
Post-high school	15 (83.3)	18 (20)		9 (50)	18 (20)	
Occupation						
Working	4 (67)	6 (7)	0.120	5 (71)	7 (8)	**0.04**
Housewife	62 (73)	85 (93)		35 (42)	83 (92)	
Monthly income per capita						
Up to 1000	21 (70)	30 (33)	0.085	11 (40)	28 (31)	0.074
1001–2000	14 (67)	21 (23)		12 (43)	28 (31)	
2001 or more	31 (78)	40 (44)		16 (47)	34 (38)	
Contraception usage						
Yes	50 (79.4)	63 (69)	** 0.001**	21 (53.8)	39 (43)	**0.041**
No	16 (57.1)	28 (31)		20 (39.2)	51 (57)	
